# Efficacy of hyperbaric oxygen combined with escitalopram in depression and its effect on cognitive function

**DOI:** 10.12669/pjms.37.4.3993

**Published:** 2021

**Authors:** Kun Mi, Qiang Guo, Bao-yan Xu, Man Wang, Hao Bi

**Affiliations:** 1 Kun Mi, The Second Department of Affective Disorders, The Sixth People’s Hospital of Hebei Province, Baoding, Hebei, 071000, P.R. China; 2 Qiang Guo, Department of Thoracic Surgery, Affiliated Hospital of Hebei University, Baoding, Hebei 071000, P.R. China; 3 Bao-yan Xu, The Second Department of Affective Disorders, The Sixth People’s Hospital of Hebei Province, Baoding, Hebei, 071000, P.R. China; 4 Man Wang, The Second Department of Affective Disorders, The Sixth People’s Hospital of Hebei Province, Baoding, Hebei, 071000, P.R. China; 5 Hao Bi, The Second Department of Affective Disorders, The Sixth People’s Hospital of Hebei Province, Baoding, Hebei, 071000, P.R. China

**Keywords:** Depression, Efficacy, Escitalopram, Hyperbaric oxygen

## Abstract

**Objective:**

To investigate the efficacy of hyperbaric oxygen (HBO) combined with escitalopram in patients with depression and its effect on cognitive function.

**Methods:**

From 2016 to 2018, seventy patients with depression aged 18-65 years treated in Affiliated Hospital of Hebei University were selected. Seventy patients with depression meeting the diagnostic criteria of ICD-10 were selected and randomly divided into control group and observation group using a random number table, with 35 patients in each group. The control group was treated with escitalopram, while the observation group was additionally treated with HBO on this basis. The patients were assessed using the Hamilton Depression Scale (HAMD) and Montreal Cognitive Assessment Scale (MoCA) before treatment and two, four and six weeks after treatment.

**Results:**

Two weeks after treatment, HAMD score showed a statistically significant difference between the two groups (*P* < 0.05). No statistically significant differences were found in HAMD score between the two groups four and six weeks after treatment (*P* > 0.05). Four and six weeks after treatment, MoCA score presented statistically significant differences between the two groups (*P* < 0.05).

**Conclusion:**

Escitalopram combined with HBO in the treatment of depression presents rapid efficacy and a certain effect in improving cognitive function.

## INTRODUCTION

Depression, often called “emotional cold”^1^, is a synthetical disease mainly characterized by persistent low spirits which can be obviously felt. At present, depression has become a major mental disease with high incidence, increasing economic and social burden and high risk of recurrence^2^, which is greatly harmful to patients themselves, their families and society. The cure rate of depression is low. The current antidepressant medication is only effective for some patients, and the recovery rate is relatively low. Moreover, the slow action of antidepressant drugs is a difficult problem in clinical practice. The poor efficacy after antidepressant medication may be manifested in residual symptoms and cognitive impairment. Continuous therapy with simple antidepressant drugs cannot always achieve the ideal effect. Therefore, it is particularly important to explore antidepressant therapy with quick action, good efficacy and improvement in cognitive function. In this study, antidepressant drug combined with hyperbaric oxygen (HBO) was used to treat patients with depression, and the results are reported as follows:

## METHODS

From 2016 to 2018, seventy patients with depression aging 18-65 years treated in Affiliated Hospital of Hebei University were selected. The Hamilton Depression Scale (HAMD) score of the patients > 18. They were divided into observation group and control group using a random number table, with 35 patients in each group. Statistical analysis showed no statistically significant differences in age, gender, course of disease or severity of disease between the two groups.

### Ethical approval

The study was approved by the Institutional Ethics Committee of Affiliated Hospital of Hebei University (No. 201802) at November 30, 2020, and written informed consent was obtained from all participants

### Inclusion criteria:

1. Patients met the diagnostic criteria of depression in ICD-10.
2. Patients had HAMD score > 18.
3. Male and female patients aged 18 to 65 years (including 18 and 65 years).
4. Patients had clear consciousness and ability to express self-feelings independently.
5. Patients and their family members signed informed consent.

### Exclusion criteria:

1. Patients with drug allergy.
2. Patients with contraindications to HBO therapy.
3. Pregnant or lactating women.
4. Patients with consciousness disorders or other severe mental disorders.
5. Patients with combined severe dysfunction of the lung, kidney, liver and heart.
6. Patients with bipolar depression.

The control group was treated with 10 mg escitalopram every morning and 20 mg escitalopram till the 6^th^ day for a total of six weeks (no other antidepressants were used during the treatment). On this basis, the observation group was additionally treated with HBO using pure oxygen at 0.12 MPa for 90 min (pressure rise for 15 minutes, continuous oxygen inhalation for 60 min after stabilization, and pressure reduce for 15 minutes), once a day, five times per week, a total of six weeks.

### Assessment basis

The severity of depression and cognitive function of the two groups were assessed before treatment and two, four and six weeks after treatment using the HAMD and Montreal Cognitive Assessment Scale (MoCA), respectively.

### Assessment criteria the severity of depression was assessed using the HAMD

HAMD score ≤ 7, non-depression; HAMD score = 8-17, mild depression; HAMD score = 18-24, moderate depression; HAMD score > 24, severe depression. According to the reduction in HAMD score, the deduction rate was determined: deduction rate of HAMD score ≥ 75%, cured; deduction rate of HAMD score ≥ 50%, significantly effective; deduction rate of HAMD score ≥ 25%, effective; deduction rate of HAMD score < 25%, invalid (deduction rate of HAMD score before and after intervention).

### Assessment of cognitive function

The degree of mild cognitive impairment was rapidly screened using the MoCA. This scale was modified based on cognitive item setting and scoring criteria of the Mini Mental State Examination (MMSE). The cognitive aspects of the assessment were constantly modified through clinical application, comprehensively covering 8 aspects: visual space and executive function, naming, memory, attention, language, abstract thinking, delayed recall and orientation. This scale has high sensitivity and specificity to patients with mild cognitive impairment, and can assess cognitive impairment in a short time. It is suitable for cognitive function assessment in patients with depression.

### Laboratory examinations

Routine blood and urine tests, liver and kidney function tests, glucose test, electrolyte test and electrocardiography (ECG) were performed before treatment and 2, 4 and 6 weeks after treatment.

### Statistical analysis

SPSS 19.0 was used for data analysis. The measurement data were expressed as mean ± standard deviation (`X ± S). Inter-group comparison was conducted using the t test or t-test. The enumeration data were compared with the chi-square test. *P* < 0.05 was considered as statistically significant.

## RESULTS

In the control group, one patient was lost to follow-up and two withdrew voluntarily, and 32 patients completed the follow-up. In the observation group, two patients were lost to follow-up one withdrew voluntarily, and 32 patients completed the follow-up. Statistical analysis was performed using the data of patients with complete follow-up. There was no statistically significant difference in HAMD score between the two groups before treatment.

Two weeks after treatment, HAMD scores of the two groups were lower than those before treatment (*P* < 0.05), and the HAMD score of the observation group was significantly different from that of the control group (*P* < 0.05). Four and 6 weeks after treatment, HAMD scores of the two groups were both lower than those before treatment (*P* < 0.05), but no statistically significant differences were found in HAMD score between the observation group and the control group (*P* > 0.05), as shown in Table-I.

**Table-I:** Comparison in HAMD score between two groups before and after treatment (`X ± S)

Group	N	HAMD score			

		Before treatment	Two weeks after treatment	Four weeks after treatment	Six weeks after treatment
Control group	32	23.56 ± 4.26	18.21 ± 3.46	12.36 ± 4.72	8.06 ± 5.39
Observation group	32	23.98 ± 4.52	16.4 ± 3.12	12.12 ± 4.02	8.03 ± 3.97
t		0.38	2.21	0.22	0.03
P		>0.05	<0.05	>0.05	>0.05

Cognitive function (MoCA score) before and after treatment Four and six weeks after treatment, MoCA scores of the two groups reduced compared with those before treatment (*P* < 0.01), and MoCA score showed statistically significant differences between the observation group and the control group (*P* < 0.05), as seen in Table-II.

**Table-II:** Cognitive function assessment (MoCA score) between two groups before and after treatment (`X ± S).

Group	N	MoCA score			

		Before treatment	Two weeks after treatment	Four weeks after treatment	Six weeks after treatment
Control group	32	13.34 ± 3.25	15.28 ± 4.24	20.32 ± 3.58	24.32 ± 3.58
Observation group	32	13.52 ± 3.56	17.10 ± 4.98	24.32 ± 4.67	26.21 ± 2.47
t		0.21	1.56	3.85	2.46
P		>0.05	>0.05	<0.05	<0.05

## DISCUSSION

At present, the treatment of depressive disorders is mainly drug therapy. Selective serotonin reuptake inhibitors have become the first-line antidepressant drugs because of their outstanding efficacy, high safety and good tolerance.^3^-^6^ Escitalopram oxalate is a highly selective 5-HT uptake inhibitor for the treatment of depression.^7^,^8^ Among them, escitalopram has a higher selectivity to 5-HT, can significantly alleviate depression and anxiety of patients, and is relatively safer. Cognitive dysfunction is a common clinical symptom of patients with depression.^9^,^10^ It has been found that cognitive impairments such as impaired executive function, inattention, memory decay and delayed reaction time are the main manifestations of some patients. The cognitive dysfunction in depressive disorders may be related to the decrease of dopamine function in prefrontal cortex and the relative enhancement of central 5-HT function.^11^ Although the symptoms of patients with depression are relieved after intervention with antidepressants, their social function damage always continues, which is closely related to the incomplete recovery of cognitive function.^12^ Therefore, increasing studies have focused on the treatment of cognitive impairment, and have shown certain advantages.^13^ However, there is still no better way to improve cognitive function.

HBO therapy can rapidly improve the hypoxic state of the brain by inhaling pure oxygen, reduce the production of necrotic brain cells, strengthen the recovery of damaged brain cells, protect the survival of neurons in the brain, and also promote the formation of new capillaries, accelerate the establishment of collateral circulation and axonal sprouting at the brain damage site, it has neuroprotective effect.^14^,^15^ HBO therapy can achieve good efficacy in hypoxic diseases, ischemic diseases or a series of diseases caused by hypoxia and ischemia.^16^-^19^ Therefore, HBO is clinically used in combination therapy to treat relevant diseases. Thomas has reported that cerebral blood flow in the anterior and posterior regions of the left and right hemispheres of patients with depression is significantly lower than that in patients with simple depressive disorders, and the decrease in left frontal blood flow is more obvious.^20^

### Limitations of the study

HBO combined with escitalopram can act earlier in depressive patients and effectively improve their cognitive function. Therefore, it is worthy of clinical application, but its long-term efficacy remains to be further observed.

## CONCLUSION

The results of this study showed that escitalopram combined with HBO in the treatment of depression-induced cognitive dysfunction presented significant clinical efficacy, and acted earlier than single application of escitalopram. The results of HAMD assessment further suggested that the efficacy of escitalopram combined with HBO was superior to escitalopram alone in the treatment of depression. Escitalopram combined with HBO therapy gives full play to their both therapeutic advantages.

### Authors’ Contributions:

**KM and** **QG** designed this study and prepared this manuscript, and are responsible and accountable for the accuracy or integrity of the work.

**BX and** **MW** collected and analyzed clinical data.

**HB** significantly revised this manuscript.

## References

[1] Li LX, Zhang YB, Gao YH, Zhang M, Zhou S (2013). Latent growth curve modeling for improvement of clinical symptoms on depression. Zhonghua Liu Xing Bing Xue Za Zhi.

[2] Perlman K, Benrimoh D, Israel S, Rollins C, Brown E, Tunteng JF (2019). A systematic meta-review of predictors of antidepressant treatment outcome in major depressive disorder. J Affect Disord.

[3] Cipriani A, Furukawa TA, Salanti G, Chaimani A, Atkinson LZ, Ogawa Y (2018). Comparative Efficacy and Acceptability of 21 Antidepressant Drugs for the Acute Treatment of Adults with Major Depressive Disorder:A Systematic Review and Network Meta-Analysis. Focus.

[4] Glassman AH, O'Connor CM, Califf RM (2002). Sertraline Antidepressant Heart Attack Randomized Trial (SADHEART) Group. Sertraline treatment of major depression in patients with acute MI or unstable angina. JAMA.

[5] Berkman LF, Blumenthal J, Burg M, Carney RM, Catellier D, Cowan MJ (2003). Enhancing Recovery in Coronary Heart Disease Patients Investigators (ENRICHD). Effects of treating depression and low perceived social support on clinical events after myocardial infarction:the Enhancing Recovery in Coronary Heart Disease Patients (ENRICHD) randomized trial. JAMA.

[6] Lesperance F, Frasure-Smith N, Koszycki D, Laliberte MA, Zyl LT, Baker B (2007). CREATE Investigators. Effects of citalopram and interpersonal psychotherapy on depression in patients with coronary artery disease:the Canadian Cardiac Randomized Evaluation of Antidepressant and Psychotherapy Efficacy (CREATE) trial. JAMA.

[7] Wang J, Pan GQ (2020). Clinical effect of low frequency repetitive transcranial magnetic stimulation combined with fasudil in the treatment of acute cerebral infarction. J China Prescription Drug.

[8] Zhu HC, Ge HM, Zhao H (2020). Effects of escitalopram oxalate combined with lamotrigine on mental state and neurological function in epilepsy comorbid depression patients after stroke. J Int Psychiatry.

[9] Ulbricht CM, Dumenci L, Rothschild AJ, Lapane KL (2016). Changes in depression subtypes for women during treatment with citalopram:a latent transition analysis. Arch Womens Ment Health.

[10] Medrano-Martinez P, Ramos-Platón MJ (2016). Cognitive and emotional alterations in chronic insomnia. Rev Neurol.

[11] Steiner AJ, Boulos N, Wright SM, Mirocha J, Smith K, Lopez E (2017). Major Depressive Disorder in Patients With Doctoral Degrees:Patient-reported Depressive Symptom Severity, Functioning, and Quality of Life Before and After Initial Treatment in the STAR*D Study. J Psychiatr Pract.

[12] Baune BT, Li X, Beblo T (2013). Shortand longterm relationships between neurocognitive performance and general function in bipolar disorder. J Clin Exp Neuropsychol.

[13] Pan Z, Park C, Brietzke E, Zuckerman H, Rong C, Mansur RB (2019). Cognitive impairment in major depressive disorder. CNS Spectr.

[14] Neretin V, Lobov MA, Kotov SV (1990). Hyperbaric oxygenation in comprehensive treatment of Parkinsonism. Neurosci Behav Physiol.

[15] Pan X, Chen C, Huang J, Wei H, Fan Q (2015). Neuroprotective effect of combined therapy with hyperbaric oxygen and madopar on 6-hydroxydopamineinduced Parkinson's disease in rats. Neurosci Lett.

[16] Babchin A, Levich E, Melamed M D Y, Sivashinsky G (2011). Osmotic phenomena in application for hyperbaric oxygen treatment. Colloids Surf B Biointerfaces.

[17] Feng JJ, Li YH (2017). Effects of hyperbaric oxygen therapy on depression and anxiety in the patients with incomplete spinal cord injury (a STROBE-compliant article). Medicine.

[18] Al-Waili NS, Butler GJ, Beale J, Abdullah MS, Hamilton RW, Lee BY (2005). Hyperbaric oxygen in the treatment of patients with cerebral stroke, brain trauma, and neurologic disease. Adv Ther.

[19] Li Q, Li J, Zhang L, Wang B, Xiong L (2007). Preconditioning with hyperbaric oxygen induces tolerance against oxidative injury via increased expression of heme oxygenase-1 in primary cultured spinal cord neurons. Life Sci.

[20] Wang Y, Liu X, Li P, Zhou H, Yang L, Zheng L (2018). Regional Cerebral Blood Flow in Mania:Assessment Using 320-Slice Computed Tomography. Front Psychiatry.

